# Effects of Cryoprotectant Concentration and Exposure Time during Vitrification of Immature Pre-Pubertal Lamb Cumulus–Oocyte Complexes on Nuclear and Cytoplasmic Maturation

**DOI:** 10.3390/ani14162351

**Published:** 2024-08-14

**Authors:** Letizia Temerario, Nicola Antonio Martino, Monika Bennink, Agnes de Wit, Sipke Joost Hiemstra, Maria Elena Dell’Aquila, Julie Lamy

**Affiliations:** 1 Department of Biosciences, Biotechnology & Environment, University of Bari Aldo Moro, Strada per Casamassima km 3, 70010 Valenzano, Italy; nicola.martino@uniba.it (N.A.M.); mariaelena.dellaquila@uniba.it (M.E.D.); 2 Animal Breeding and Genomics, Wageningen University & Research, 6700 AH Wageningen, The Netherlands; monika1.bennink@wur.nl (M.B.); agnes.dewit@wur.nl (A.d.W.); sipkejoost.hiemstra@wur.nl (S.J.H.); julie.lamy@wur.nl (J.L.)

**Keywords:** sheep, endangered breed, conservation, oocyte, vitrification, cryoprotectant, in vitro maturation, oocyte bioenergetic-oxidative status

## Abstract

**Simple Summary:**

In the last years, many domesticated and wild animal species, as well as local and transboundary breeds, have been threatened with genetic depletion and extinction. The application of conservation strategies to sheep breeds is necessary to safeguard the productive characteristics but also the historical and cultural value that they exert on their territory and inhabitants. Despite many advances that have been made in gamete cryopreservation, in vitro embryo production (IVEP) and juvenile in vitro production (JIVET), the efficiency of these technologies is still low, and the application is restricted in the ovine species. In the present study, a high concentration-rapid exposure (HC-RE) and a low concentration-slow exposure (LC-SE) vitrification protocol, using dimethyl sulfoxide (DMSO) and ethylene glycol (EG) as permeating cryoprotectants (CPAs), were applied to pre-pubertal lamb immature cumulus–oocyte complexes (COCs) and evaluated on nuclear and cytoplasmic parameters after in vitro maturation (IVM). Slightly more encouraging results were observed with the LC-SE vitrification protocol, leading to the hypothesis that low CPA concentrations in association with prolonged exposure times could be more promising to pursue in order to improve pre-pubertal lamb immature COC vitrification.

**Abstract:**

Oocyte vitrification allows for the storing of endangered breed female gametes. Cryoprotectant (CPA) concentration and exposure time should ensure cell protection with minimal toxicity. In the present study, a high concentration-rapid exposure (HC-RE) and a low concentration-slow exposure (LC-SE) vitrification protocol, using dimethyl sulfoxide (DMSO) and ethylene glycol (EG) as permeating CPAs, were evaluated on meiotic competence and bioenergetic-oxidative status of pre-pubertal lamb immature COCs after in vitro maturation (IVM). For each protocol, COCs vitrified through a traditional protocol and fresh ones were used as controls. Both protocols allowed COC morphology preservation after vitrification-warming (V-W) and cumulus expansion after IVM. The maturation rate (7% and 14%) was comparable to the vitrified control (13% and 21%) but not satisfactory compared to fresh ones (58% and 64%; *p* < 0.001). The rate of mature oocytes displaying a perinuclear/subcortical (P/S) mitochondrial distribution pattern, an index of cytoplasmic maturity, was comparable between vitrified and fresh oocytes. The LC-SE vitrification protocol did not affect quantitative bioenergetic-oxidative parameters compared to both controls whereas HC-RE protocol significantly reduced intracellular reactive oxygen species (ROS) levels, indicating cell viability loss. In conclusion, to improve pre-pubertal lamb immature COC vitrification, the combination of low CPA concentrations with prolonged exposure time could be more promising to investigate further.

## 1. Introduction

The application of reproductive biotechnologies to small ruminants and in particular to sheep is not only interesting for meat, milk and wool productive characteristics but also for the historical and cultural value that many local breeds exert on their territory and inhabitants [1,2,3,4]. Currently, research with a sheep model is performed by using ovaries from both adult ewes and pre-pubertal lambs, with high and low developmental competence, respectively [5,6,7,8,9,10,11]. In particular, oocytes retrieved from juvenile animals, widely available due to lamb meat consumption, allow researchers to rescue female germplasm from slaughterhouses without interference with farm productive and reproductive activities [1], to obtain more COCs than from adult ewe ovaries and to increase the genetic gain by shortening the generation interval [12,13,14]. Despite all the progress that has been made in gamete cryopreservation, in vitro embryo production (IVEP) and juvenile in vitro production (JIVET), the efficiency of these technologies is still low and the application is restricted in the ovine species [15,16,17]. Therefore, the improvement of reproductive biotechnologies is necessary to ensure a wider propagation of valuable genes, facilitate the distribution of superior genotypes and contribute to the preservation of endangered breeds in the ovine species [16].

Oocyte cryopreservation is a valuable tool to safeguard the female germplasm of high value subjects and breeds threatened by the risk of extinction [1,18,19,20,21]. Indeed, frozen samples can be stored in liquid nitrogen, within germplasm gene banks, for a potentially unlimited time and fertilized with selected semen samples, when necessary, to obtain live offspring in support of in vivo conservation strategies, against current and future calamities [22]. In particular, with the aim to preserve animal biodiversity, immature cumulus–oocyte complexes (COCs) cryopreservation, through vitrification technique, allows for collection and storage of a large number of female gametes in field conditions, directly in farms and slaughterhouses, often located in areas lacking laboratories, without the need to induce ovarian hyperstimulation and allowing for scheduling of the day of in vitro maturation (IVM) and subsequent procedures in specialized and adequate laboratories [1,18]. Moreover, immature COCs are considered less sensitive to vitrification compared to mature oocytes as the genetic material which is protected within the nuclear envelope, thus avoiding chromosome and microtubule damage [23,24]. However, female gametes are poorly represented in the collections of most European animal gene banks, due to the greater difficulty of recovery and the lower successful outcome after cryopreservation, compared to the male counterpart [22]. Indeed, during vitrification and warming procedures, oocytes are challenged by ice crystal formation, cryoprotectant (CPA) toxicity, water and CPA molecule movements across the plasma membrane as well as temperature fluctuations [25], thus leading to chemical, mechanical, osmotic and thermal stress damage [25]. Subsequently, COCs may undergo morphological changes [18], physical/functional detachment of cumulus cells [18], chromosome abnormalities [26], altered mitochondrial function and distribution and oxidative stress [27,28,29,30], therefore limiting oocyte viability and developmental competence after vitrification.

Nowadays, the most currently used vitrification protocol for oocyte vitrification is a two-step procedure involving two solutions with increasing CPA concentrations: equilibration solution (ES) and vitrification solution (VS) [31]. The first step aims to slowly replace intracellular water by CPAs until equilibrium is reached, using low concentrations of penetrating CPAs combined with prolonged exposure times. The second step is needed to quickly dehydrate the cells with a high concentration of penetrating and non-penetrating CPAs, thus avoiding intracellular ice formation when plunging in liquid nitrogen [32]. Dimethyl sulfoxide (DMSO) and ethylene glycol (EG) are among the most commonly used penetrating CPAs during the vitrification procedure. These molecules, characterized by low molecular weight and amphiphilic properties, are able to replace intracellular water, allowing dehydration, thus reducing ice crystal formation [25,26]. Oocyte permeability to these CPAs is influenced by concentration [33], exposure time [34,35], temperature [34], meiosis stage [36] and finally by structural and physiological differences between adult and pre-pubertal oocytes [37]. Oolemma permeability tends to be higher for DMSO than for EG [38]. Moreover, it has been observed that this CPA is able to modulate the permeability of biological membranes in a concentration dependent manner. Indeed, low concentrations of DMSO (5% *v*/*v*) reduce membrane thickness therefore increasing its permeability. At normally used concentrations (10% *v*/*v*), DMSO induces water pore formation that favors water replacement with CPA molecules. However, DMSO can also disintegrate phospholipid bilayers at higher and more toxic concentrations (40% *v*/*v*) [33]. Therefore, CPA exposure time should be long enough to allow a sufficient dehydration but not so long to avoid cell damage and reduction in the developmental potential [39]. Finally, in correlation with the meiosis stage, it is known that immature germinal vesicle (GV) oocytes are characterized by reduced permeability coefficients to water and CPAs in comparison to in vitro matured oocytes [36,38], with higher permeability to low concentrations of EG [36].

We hypothesized that a longer exposure to a lower concentration of permeating CPAs (DMSO/EG) would allow better entrance of CPAs inside immature COCs and therefore a better vitrification process and outcome. The aim of the present study was to analyze the effect of CPA concentration and exposure time during vitrification of pre-pubertal lamb immature COCs. To test our hypothesis, two different protocols, high concentration-rapid exposure (HC-RE) and low concentration-slow exposure (LC-SE), were tested and assessed on oocyte nuclear and cytoplasmic maturation after IVM and compared to a control vitrification protocol and a fresh control not undergoing vitrification.

## 2. Materials and Methods

### 2.1. Chemicals

All chemicals for in vitro cultures and analyses were purchased from Sigma-Aldrich (Milan, Italy), unless otherwise indicated.

### 2.2. Ovary Collection and COC Recovery

Pre-pubertal lamb (younger than six months of age) ovaries were obtained from slaughtered animals subjected to routine veterinary inspection. After transport to the laboratory at room temperature (within 2 h after collection), ovaries were processed through a slicing procedure in order to collect COCs in phosphate buffered saline (PBS) solution inside sterile Petri dishes [1]. The follicular contents were observed under a Nikon SMZ800N stereomicroscope (Nikon, Tokyo, Japan) equipped with a transparent heating stage set up at 25 ± 2 °C. COCs exhibiting a minimum of three intact cumulus cell layers and homogenous cytoplasm were selected [1].

### 2.3. COC Vitrification and Warming

**Figure 1:**
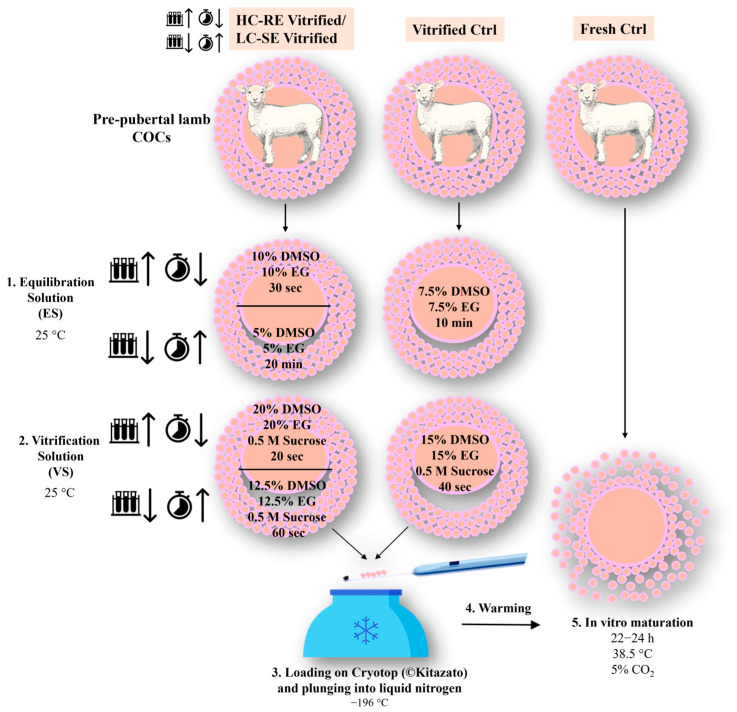
Schematic representation of the experimental design described in the present study. Pre-pubertal lamb immature COCs were vitrified through a high concentration-rapid exposure (HC-RE) or low concentration-slow exposure (LC-SE) vitrification protocol, using DMSO and EG as permeating CPAs. After warming, COCs underwent IVM. Immature COCs cryopreserved through a traditional vitrification protocol and fresh COCs directly subjected to IVM after selection were used as controls. COC = cumulus–oocyte complex; CPA = cryoprotectant; DMSO = dimethyl sulfoxide; EG = ethylene glycol; IVM = in vitro maturation.

For each experiment, pre-pubertal lamb immature COCs were randomly assigned to three different groups: (1) HC-RE or LC-SE vitrification protocol, (2) traditional vitrification protocol and (3) fresh control group not undergoing vitrification. Figure 1 shows a schematic representation of the experimental design described in the present study.

In the HC-RE protocol, COCs were exposed for 30 s to the equilibration solution (ES), in 300 μL drops, containing 10% (*v*/*v*) EG and 10% (*v*/*v*) DMSO dissolved in base medium (BM), consisting of 20% (*v*/*v*) fetal calf serum (FCS) in Hepes-buffered TCM 199. After equilibration, the oocytes were exposed to vitrification solution (VS), in 300 μL drops, containing 20% (*v*/*v*) EG, 20% (*v*/*v*) DMSO and 0.5 mol/L sucrose dissolved in BM. Groups of 5 COCs were loaded onto a Cryotop (©Kitazato, Shizuoka, Japan) vitrification device with a minimum volume (e.g., <0.1 μL) and plunged quickly into liquid nitrogen. The total time between the start of exposure to VS and plunging into liquid nitrogen was 20 s.

In the LC-SE vitrification protocol, COCs were incubated for 20 min in 300 μL drops of ES containing 5% (*v*/*v*) EG and 5% (*v*/*v*) DMSO and dissolved in BM. After equilibration, the oocytes were placed into 300 μL drops of VS containing 12.5% (*v*/*v*) EG, 12.5% (*v*/*v*) DMSO and 0.5 mol/L sucrose dissolved in BM. Groups of 5 COCs were loaded onto Cryotop (©Kitazato, Shizuoka, Japan) with a minimum volume (e.g., <0.1 μL) and plunged quickly into liquid nitrogen. The total time between the start of exposure to VS and plunging into liquid nitrogen was 60 s.

Each tested protocol was compared with a traditional vitrification protocol characterized by intermediate CPA concentration and exposure duration [1]. Briefly, selected immature COCs were incubated for 10 min in 300 μL drops of ES containing 7.5% (*v*/*v*) EG and 7.5% (*v*/*v*) DMSO and dissolved in BM. After equilibration, the oocytes were placed into 300 μL drops of VS containing 15% (*v*/*v*) EG, 15% (*v*/*v*) DMSO and 0.5 mol/L sucrose dissolved in BM. Groups of 5 COCs were loaded onto Cryotop (©Kitazato, Shizuoka, Japan) with a minimum volume (e.g., <0.1 μL) and plunged quickly into liquid nitrogen. The total time between the start of exposure to VS and plunging into liquid nitrogen was 40 s. In all three protocols, vitrification media were used at room temperature in order to counteract CPA toxicity.

For all protocols, COCSs were warmed as follows: vitrification devices were submerged into the warming solution (WS) consisting of 1 mol/L sucrose dissolved in BM at 38.5 °C for 1 min. COCs were transferred to 300 μL drops of dilution solution (DS) containing BM plus sucrose 0.5 mol/L for 3 min and washed in 300 μL drops of BM for 5 min at 25 ± 2 °C.

### 2.4. COC Morphology Assessment after Vitrification-Warming

After vitrification-warming (V-W), COC morphology was assessed as preserved or not depending on whether the cumulus appeared complete, compact and multilayered or partially/completely denuded.

### 2.5. In Vitro Maturation (IVM)

IVM was performed as reported previously in pre-pubertal lambs [1]. IVM medium was prepared based on TCM-199 medium with Earle’s salts, buffered with 5.87 mmol/L HEPES and 33.09 mmol/L sodium bicarbonate and supplemented with 0.1 g/L L-glutamine, 2.27 mmol/L sodium pyruvate, calcium lactate pentahydrate (1.62 mmol/L Ca^2+^, 3.9 mmol/L lactate), 50 μg/mL gentamicin, 20% (*v*/*v*) fetal calf serum (FCS), 10 μg/mL of porcine follicle stimulating hormone and luteinizing hormone (FSH/LH; Pluset^®^, Calier, Barcelona, Spain) [40] and 1 μg/mL 17β estradiol [1]. IVM medium was pre-equilibrated for 1 h under 5% CO_2_ in air at 38.5 °C. In each experiment, groups of 20–25 COCs were placed in four-well dishes (Nunc Intermed, Roskilde, Denmark) containing 400 μL of IVM culture medium per well covered with pre-equilibrated lightweight mineral oil and cultured for 22–24 h at 38.5 °C under 5% CO_2_ in air.

### 2.6. Cumulus Expansion Assessment and Oocyte Denuding

After IVM, COCs were classified as expanded or compact depending on whether they presented discontinuous or continuous edges of cumulus cells. For oocyte denuding, COCs were incubated in TCM-199 with 20% FCS containing 80 IU hyaluronidase/mL and aspirated inside and outside of pipettes. Denuded oocytes were assessed for meiosis stage, and mature ones were evaluated for bioenergetic/oxidative status.

### 2.7. Oocyte Mitochondria and ROS Staining

To detect and localize mitochondria and reactive oxygen species (ROS), oocytes were stained with MitoTracker Orange CMTM Ros (Thermo Fisher Scientific, Waltham, MA, USA) and 2,7-dichlorodihydrofluorescein diacetate (H_2_DCF-DA), and fixed in 4% paraformaldehyde (PFA) solution in PBS [1,41].

### 2.8. Oocyte Nuclear Chromatin Evaluation

After fixation, oocytes were stained with 2.5 μg/mL Hoechst 33,258 in 3:1 (*v*/*v*) glycerol/PBS. Oocytes were mounted on microscope slides and examined through an epifluorescence microscope (Nikon Eclipse TE300; Nikon Instruments, Florence, Italy) equipped with the objective Nikon Plan Fluor 40×/NA 0.75 and a B-2A (346 nm excitation/460 nm emission) filter. According to the meiosis stage, oocytes were classified as germinal vesicle (GV), metaphase to telophase I (MI to TI) and MII with the first polar body (PB) extruded. Oocytes showing multipolar meiotic spindle, irregular chromatin distribution or chromatin absence were considered abnormal [1].

### 2.9. Mitochondrial Distribution Pattern and Intracellular ROS Localization Assessment

Mature oocytes were observed using a Nikon C1/TE2000-U laser scanning confocal microscope (Nikon Instruments, Florence, Italy) equipped with the Apo 60×/NA 1.40 Nikon Plan objective in oil immersion. A 543 nm helium/neon laser and a G-2A filter were used to detect the MitoTracker Orange CMTM Ros (551 nm excitation and 576 nm emission). A 488 nm argon ion laser and a B-2A filter were used to detect dichlorofluorescein (DCF) (495 nm excitation and 519 nm emission). Oocytes were observed in 25 optical sections with a step size of 0.45 μm, thus allowing 3D distribution analysis. The mitochondrial distribution pattern was evaluated as “perinuclear and subcortical (P/S)”, index of cytoplasmic maturity, “finely granular”, typical of immature oocytes and “abnormal”, with irregular mitochondria distribution [1]. Concerning intracellular ROS localization, oocytes with intracellular ROS diffused throughout the cytoplasm, together with areas/sites of mitochondria/ROS overlapping, were considered viable.

### 2.10. Quantification of Bioenergetic/Oxidative Parameters

In each individual oocyte, MitoTracker and DCF fluorescence intensities and the Manders’ overlap coefficient [42], indicating the extent of mitochondria/ROS colocalization, were measured at the equatorial plane using the EZ-C1 Gold Version 3.70 image analysis software platform for a Nikon C1 confocal microscope. A circular area was drawn around the ooplasm for the quantification analysis. The fluorescence intensity within the scanned area (512 × 512 pixels) was recorded and 16-bit images were obtained. Mitochondrial membrane potential (ΔΨ) and intracellular ROS concentrations were recorded as the fluorescence intensity emitted by each probe and expressed as arbitrary densitometric units (ADUs). Sample signals were expressed as percentages of the signal of the sample used as a control (fresh ctrl). Variables related to fluorescence intensity, such as laser energy, signal detection (gain) and pinhole size, were maintained at constant values for all measurements. In mitochondria/ROS colocalization analysis, threshold levels were kept constant at 10% of the maximum pixel intensity, for all measurements.

### 2.11. Statistical Analysis

The rates of COC preserved morphology, cumulus expansion, oocyte nuclear chromatin configurations and mitochondria distribution patterns, and the quantification data of bioenergetic/oxidative parameters (ΔΨ, intracellular ROS levels and mitochondria/ROS colocalization) were compared using one-way analysis of variance (ANOVA) followed by Tukey’s multiple comparison test. Data were analyzed with GraphPad Prism (Software version 5.03, San Diego, CA, USA). Differences were considered statistically significant at *p* < 0.05. The statistical charts were prepared using Microsoft Excel (Microsoft Office 365, Redmond, WA, USA).

## 3. Results

### 3.1. HC-RE Vitrification Protocol Preserved COC Morphology

**Figure 2:**
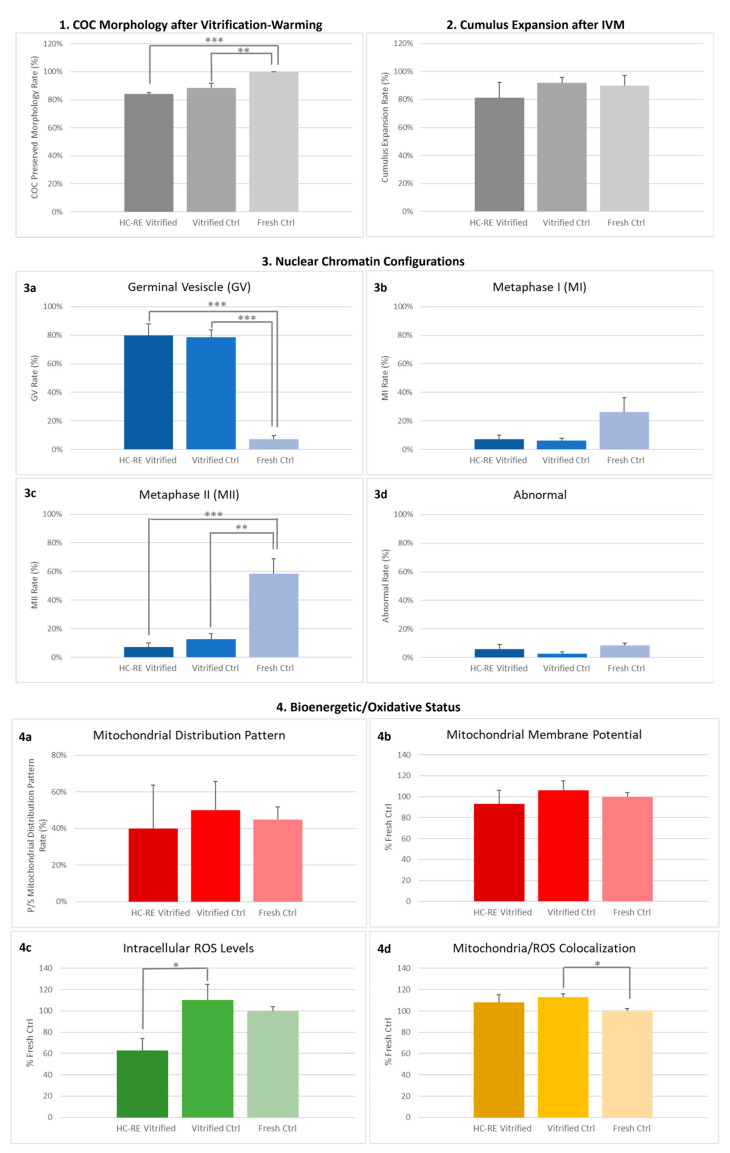
Effect of HC-RE vitrification protocol in pre-pubertal lamb COCs on (**1**) COC preserved morphology rate after vitrification and warming. A total of 75–89 COCs were evaluated per condition in 4 replicates. Each bar represents mean + SEM, (**2**) cumulus expansion rate after IVM. A total of 75–89 COCs were evaluated per condition in 4 replicates. Each bar represents mean + SEM, (**3**) nuclear chromatin configuration rate after IVM. A total of 70–84 oocytes were evaluated per condition in 4 replicates. Each bar represents mean + SEM and (**4**) qualitative (perinuclear/subcortical P/S mitochondrial distribution pattern) and quantitative (mitochondrial membrane potential, intracellular reactive oxygen species ROS levels and mitochondria/ROS colocalization) bioenergetic/oxidative parameters in MII oocytes obtained in HC-RE vitrified, vitrified and fresh ctrl (5, 10 and 49 oocytes, respectively). For quantitative parameters, each bar represents mean presented as a percentage of the signal of fresh ctrl + SEM. One-way analysis of variance ANOVA followed by Tukey’s multiple comparison test: * *p* < 0.05; ** *p* < 0.01; *** *p* < 0.001.

**Figure 3:**
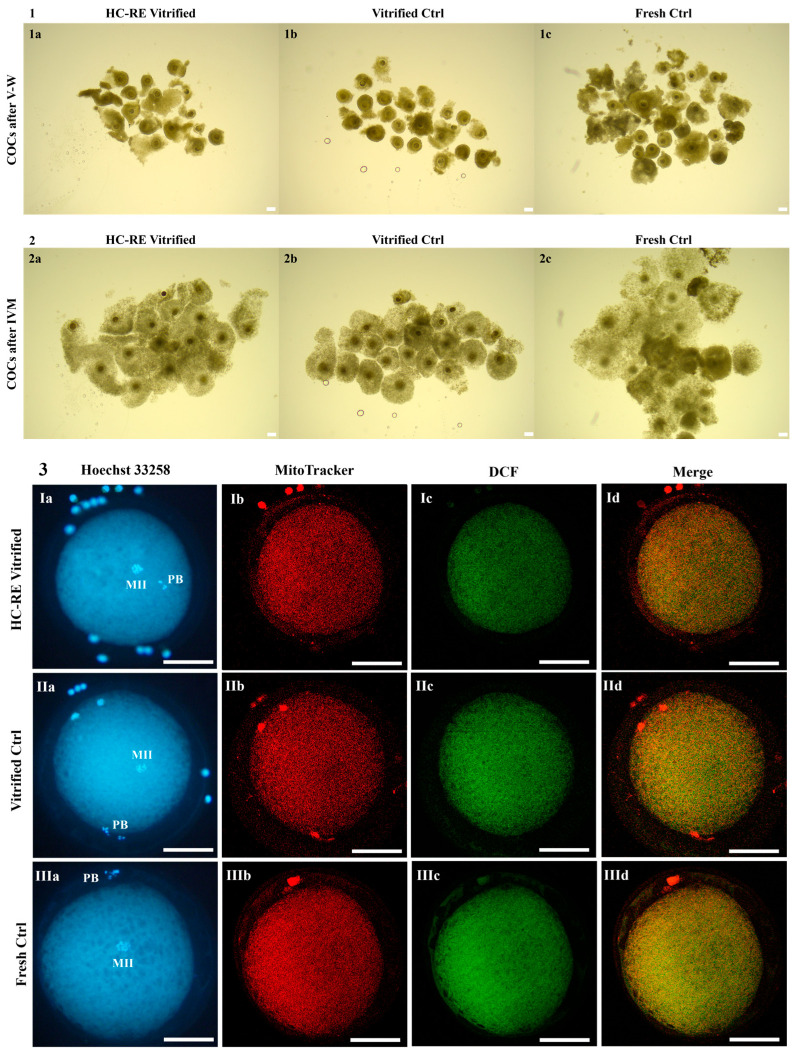
Effects of HC-RE vitrification protocol in pre-pubertal lamb COCs: (**1**) pre-pubertal lamb COCs in HC-RE vitrified, vitrified ctrl and fresh ctrl, as observed before IVM. Scale bars represent 120 µm. (**2**) Pre-pubertal lamb COCs in HC-RE vitrified, vitrified ctrl and fresh ctrl, as observed after IVM. Scale bars represent 120 µm. (**3**) Photomicrographs showing representative images of one MII oocyte obtained in HC-RE vitrified, vitrified ctrl and fresh ctrl after IVM. Corresponding epifluorescence images showing (**Ia**–**IIIa**) nuclear chromatin configuration (Hoechst 33258) and confocal images showing (**Ib**–**IIIb**) mitochondrial distribution pattern and activity (MitoTracker Orange), (**Ic**–**IIIc**) intracellular ROS localization and levels (DCF) and (**Id**–**IIId**) mitochondria/ROS colocalization (Merge). Confocal images were taken at the oocyte equatorial plane. Scale bars represent 40 µm.

The rate of COCs which showed preserved morphology after V-W, displaying complete, compact and multilayered cumuli, did not differ after the application of HC-RE vitrification protocol in comparison to ctrl protocol (Figure 2 panel 1 and Figure 3 panel 1). This parameter was significantly reduced by both vitrification protocols compared to fresh COCs (Figure 2 panel 1 and Figure 3 panel 1; *p* < 0.01 and *p* < 0.001, respectively), even if absolute values remained high at any condition.

### 3.2. HC-RE Vitrification Protocol Allowed Cumulus Expansion

After IVM, the cumulus expansion rate of COCs vitrified through HC-RE protocol did not differ compared to COCs vitrified through ctrl protocol and with fresh ones, thus indicating a positive response to the gonadotropins added in the culture medium, in any examined conditions (Figure 2 panel 2 and Figure 3 panel 2).

### 3.3. HC-RE Vitrification Protocol Reduced Oocyte Meiotic Maturation

The rate of mature oocytes, obtained after COC vitrification through both HC-RE and ctrl protocol and subsequent IVM, were significantly lower in comparison to fresh ones (*p* < 0.001 and *p* < 0.01, respectively; Figure 2 panel 3), without differences between the two vitrification groups. In both cases, corresponding higher rates of GV-stage oocytes were observed (*p* < 0.001 and *p* < 0.01, respectively; Figure 2 panel 3), demonstrating that both vitrification protocols induced severe modifications in the oocyte, thus strongly affecting the ability to resume meiosis and reach maturation.

### 3.4. HC-RE Vitrification Protocol Did Not Affect Oocyte Mitochondria Distribution Pattern

In MII oocytes obtained after IVM, qualitative and quantitative parameters of bioenergetic/oxidative status were assessed as indicators of oocyte cytoplasmic maturation and competence to undergo subsequent fertilization and development. As a qualitative parameter, the rate of mature oocytes displaying heterogeneous P/S mitochondrial distribution pattern did not vary among fresh and vitrified mature oocytes obtained after HC-RE and ctrl protocol, indicating that this parameter of cytoplasmic maturation was reached in any examined condition (Figure 2 panel 4).

### 3.5. HC-RE Vitrification Protocol Reduced Oocyte Intracellular ROS Levels

In regards to quantitative parameters, HC-RE vitrification protocol significantly reduced intracellular ROS levels in mature oocytes after IVM, in comparison to vitrified ctrl (*p* < 0.05; Figure 2 panel 4 and Figure 3 panel 3). However, values of ΔΨ and mitochondria/ROS colocalization did not differ between the two vitrification groups and fresh ctrl. The ctrl vitrification protocol induced a significant increase in mitochondria/ROS colocalization in comparison to fresh ctrl (*p* < 0.05; Figure 2 panel 4 and Figure 3 panel 3).

### 3.6. LC-SE Vitrification Protocol Preserved COC Morphology

**Figure 4:**
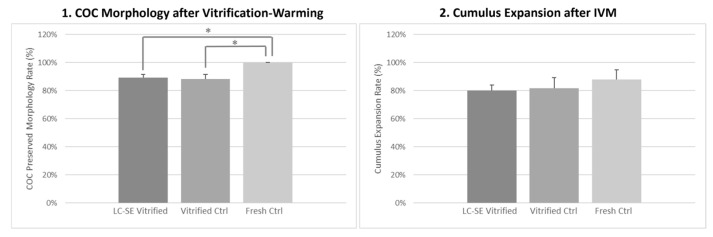

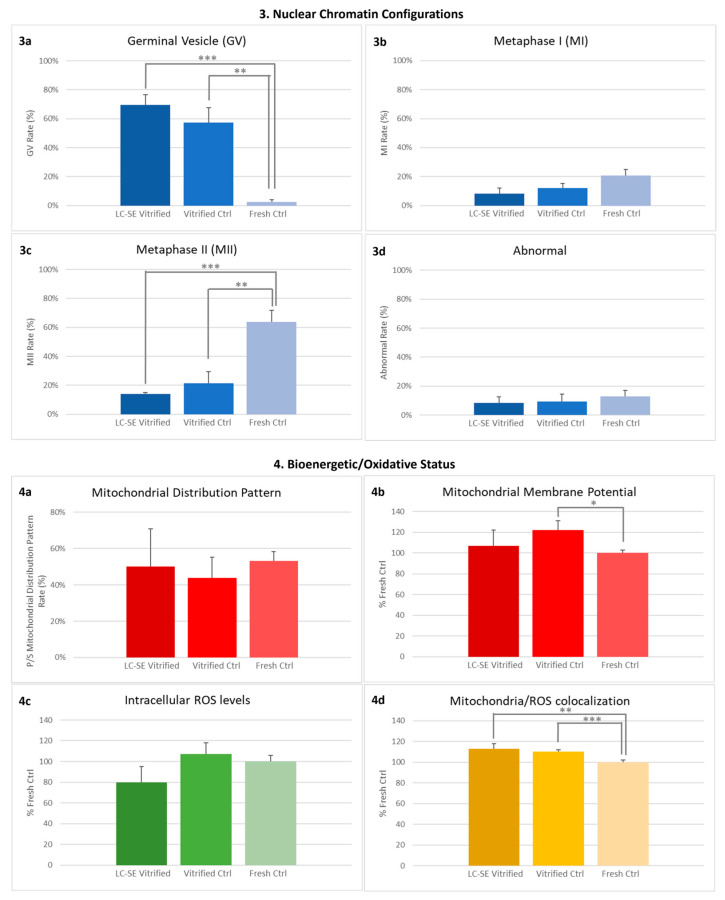
Effect of LC-SE vitrification protocol in pre-pubertal lamb COCs on (**1**) COC preserved morphology rate after vitrification and warming. A total of 75–82 COCs were evaluated per condition in 4 replicates. Each bar represents mean + SEM, (**2**) cumulus expansion rate after IVM. A total of 75–82 COCs were evaluated per condition in 4 replicates. Each bar represents mean + SEM, (**3**) nuclear chromatin configuration rate after IVM. A total of 72–77 oocytes were evaluated per condition in 4 replicates. Each bar represents mean + SEM and (**4**) qualitative (perinuclear/subcortical P/S mitochondrial distribution pattern) and quantitative (mitochondrial membrane potential, intracellular reactive oxygen species ROS levels and mitochondria/ROS colocalization) bioenergetic/oxidative parameters in MII oocytes obtained in LC-SE vitrified, vitrified and fresh ctrl (10, 16 and 48 oocytes, respectively). For quantitative parameters, each bar represents mean presented as a percentage of the signal of fresh ctrl + SEM. One-way analysis of variance ANOVA followed by Tukey’s multiple comparison test: * *p* < 0.05; ** *p* < 0.01; *** *p* < 0.001.

**Figure 5:**
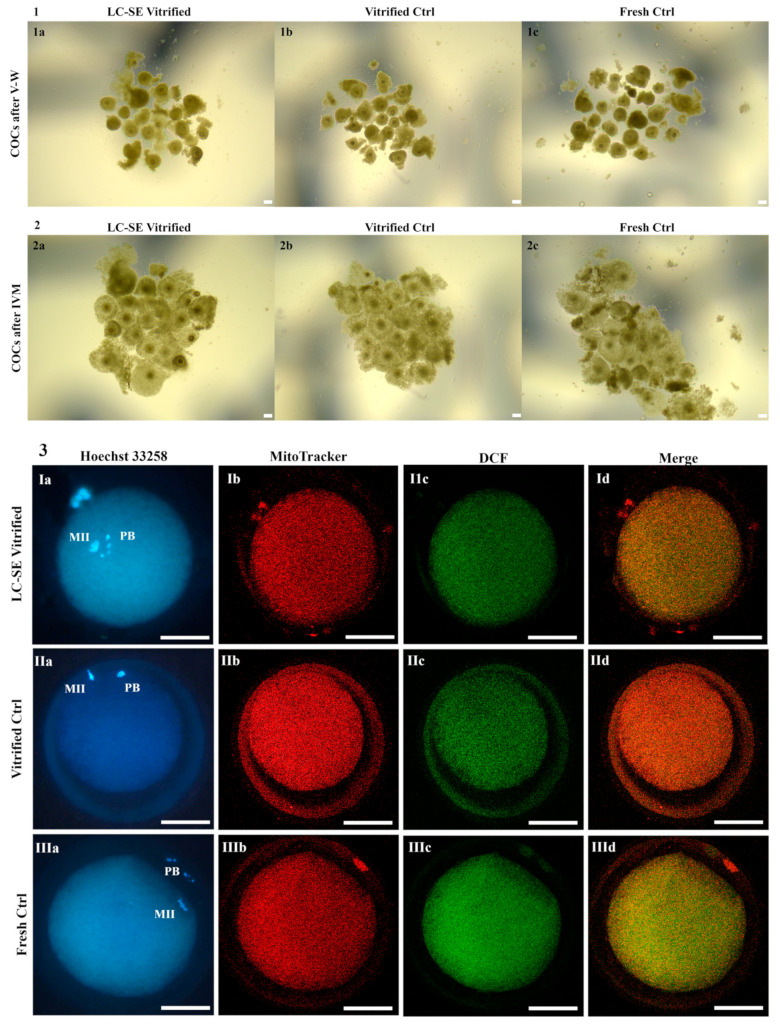
Effects of LC-SE vitrification protocol in pre-pubertal lamb COCs: (**1**) pre-pubertal lamb COCs in LC-SE vitrified, vitrified ctrl and fresh ctrl, as observed before IVM. Scale bars represent 120 µm. (**2**) Pre-pubertal lamb COCs in LC-SE vitrified, vitrified ctrl and fresh ctrl, as observed after IVM. Scale bars represent 120 µm. (**3**) Photomicrographs showing representative images of one MII oocyte obtained in LC-SE vitrified, vitrified ctrl and fresh ctrl after IVM. Corresponding epifluorescence images showing (**Ia**–**IIIa**) nuclear chromatin configuration (Hoechst 33258) and confocal images showing (**Ib**–**IIIb**) mitochondrial distribution pattern and activity (MitoTracker Orange), (**Ic**–**IIIc**) intracellular ROS localization and levels (DCF) and (**Id**–**IIId**) mitochondria/ROS colocalization (Merge). Confocal images were taken at the oocyte equatorial plane. Scale bars represent 40 µm.

The application of LC-SE vitrification protocol highlighted comparable rates of COCs showing preserved morphology at warming with the vitrification control group (Figure 4 panel 1 and Figure 5 panel 1). This parameter was significantly reduced by both vitrification protocols compared to fresh COCs (*p* < 0.05; Figure 4 panel 1 and Figure 5 panel 1) even if absolute values after both vitrification procedures were still high.

### 3.7. LC-SE Vitrification Protocol Allowed Cumulus Expansion

After IVM, the cumulus expansion rate of COCs vitrified through the LC-SE protocol did not differ compared to COCs vitrified through the ctrl protocol and with fresh ones, thus indicating a positive response to the gonadotropins added in the culture medium, in all examined conditions (Figure 4 panel 2 and Figure 5 panel 2).

### 3.8. LC-SE Vitrification Protocol Reduced Oocyte Meiotic Maturation

After COC vitrification, through both LC-SE and ctrl protocol and subsequent IVM, the rates of mature oocytes were significantly lower in comparison to fresh ones (*p* < 0.001 and *p* < 0.01, respectively; Figure 4 panel 3), with no differences between the two vitrification groups. In both cases, corresponding higher rates of GV-stage oocytes were observed (*p* < 0.001 and *p* < 0.01, respectively; Figure 4 panel 3), demonstrating that, also in this case, both protocols affected the ability of vitrified oocytes to resume meiosis and reach maturation.

### 3.9. LC-SE Vitrification Protocol Did Not Affect Oocyte Mitochondria Distribution Pattern

The rates of mature oocytes exhibiting heterogeneous P/S mitochondrial distribution pattern did not vary among fresh and vitrified oocytes, after both LC-SE and ctrl protocol, indicating that this parameter of cytoplasmic maturation was reached in experimental groups (Figure 4 panel 4).

### 3.10. LC-SE Vitrification Protocol Did Not Affect Quantitative Parameters of Bioenergetic-Oxidative Status

Values of MII oocyte ΔΨ, intracellular ROS levels and mitochondria/ROS colocalization were not affected by vitrification through LC-SE protocol in comparison to vitrified ctrl (Figure 4 panel 4 and Figure 5 panel 3). Moreover, these oocytes showed a significant increase in the mitochondria/ROS colocalization in comparison to fresh ctrl (*p* < 0.01; Figure 4 panel 4 and Figure 5 panel 3). Finally, regarding vitrified ctrl, ΔΨ and mitochondria/ROS colocalization were significantly increased in comparison to fresh ctrl (*p* < 0.05 and *p* < 0.001, respectively; Figure 4 panel 4 and Figure 5 panel 3).

## 4. Discussion

The success of immature COC vitrification is limited in most farm animal species due to structural complexity, physiological and functional characteristics. In sheep, whose oocytes show a marked sensitivity to chilling and freezing, associated with poor developmental rates [43,44,45] and no report of live births, research and applications are restricted compared to other species such as bovine [46,47,48,49], porcine [50] and equine [51,52]. A critical aspect of vitrification is represented by CPA choice, concentration and exposure time, in order to protect the oocyte during freezing as well as ensure the minimum possible toxicity [35].

In the present study, a high concentration-rapid exposure (HC-RE) and a low concentration-slow exposure (LC-SE) vitrification protocol, using DMSO and EG as penetrating CPAs, were tested against a ctrl vitrification protocol to evaluate which of the two factors, CPA concentration or exposure time, determines better protection of pre-pubertal lamb COCs after V-W, through assessment of oocyte meiotic competence and bioenergetic-oxidative status after IVM.

Although the rate of COCs with preserved morphology after V-W, displaying complete, compact and multilayered cumuli, was significantly reduced compared to COCs directly placed in IVM culture, the absolute values remained high after both vitrification procedures as well as in vitrified ctrl, with partial detachment of the cumulus cells being the most frequently observed alteration. Our data with ovine COCs are in line with previous results in the porcine species [53] and are encouraging compared to a former study in the domestic cat [54], in which oocyte shape changes and cumulus cell loss were described, among other morphological cryodamage. These differences could be due to intrinsic features of ovine COCs characterized by a greater number of cumulus cell layers compared to cat ones.

Both tested vitrification protocols allowed cumulus expansion, indicating survival, receptivity to gonadotropins and bidirectional communication between oocytes and cumulus cells, without significant differences compared to vitrified and fresh ctrl. These data represent an improvement in comparison to our previous study in pre-pubertal lambs [1], in which it was observed that vitrified COCs through the traditional protocol significantly reduced their expansion rate compared to fresh ctrl and former studies in adult sheep [55,56]. These differences may be due to the variability in vitrification device or COC intrinsic features from different sheep breeds and farming systems.

Although encouraging results of preserved morphology after V-W and cumulus expansion after IVM were found, the nuclear maturation rate was not improved by any protocol compared to vitrified ctrl and was significantly reduced in comparison to fresh COCs. These data are in line with previous studies in pre-pubertal lambs [1] and adult sheep [24,44,55,56,57,58,59], in which immature COC vitrification resulted in low rates of meiosis resumption with achievement of metaphase II stage compared to fresh COCs. The HC-RE vitrification protocol could have damaged the oocytes due to potentially toxic concentrations of CPAs associated with minimal exposure time, which did not allow the replacement of enough intracellular water with CPAs, thus leading to intracellular ice formation and impairment of the maturation process. According to the low permeability of GV oocyte membranes [36,38] and lower number of transzonal projections in pre-pubertal lamb COCs different from adult ones [37], more promising results could have been expected through prolonged exposure to less toxic concentrations of CPAs (LC-SE vitrification protocol). Intracellular ice formation could have occurred due to low amounts of CPA within the cells despite longer exposure time. Moreover, both protocols may have induced morphological and functional damage, altering the meiosis resumption pathways. Therefore, further studies are needed to identify the best, balanced combination of concentration and exposure time to improve the pre-pubertal lamb immature COC vitrification protocol.

In addition, vitrification is known to alter the proper mitochondria distribution and function with ROS overproduction, thus inducing oxidative stress with subsequent organelle damage [27,28,60]. Despite the low nuclear maturation rate, the percentage of MII oocytes showing P/S mitochondrial distribution pattern, in both tested vitrification protocols, did not differ compared to vitrified and fresh ctrl, thus indicating the achievement of cytoplasmic maturation. These data are in agreement with our previous results in pre-pubertal lambs [1], while they differ from those of a study in adult sheep in which it was observed that vitrification significantly increased the rate of oocytes with an abnormal mitochondrial distribution pattern, displaying clusters of numerous mitochondria within the cytoplasm [57], probably due to differences in the used vitrification devices.

Regarding quantitative parameters of the bioenergetic-oxidative status, the LC-SE vitrification protocol provided more encouraging results compared to the HC-RE one. Indeed, both tested protocols did not influence ΔΨ levels compared to vitrified and fresh ctrl, whereas HC-RE induced lower ROS levels compared to the other experimental conditions. The observed marked reduction in ROS levels, upon HC-RE protocol application, could indicate a loss of cell viability rather than an antioxidant effect. Indeed, ROS are molecules physiologically produced by mitochondria during cellular metabolism and generally an increase in ΔΨ in response to a stress stimulus, such as slow freezing or vitrification [61], is accompanied by a physiological increase in ROS levels. Instead, if the stress to which the cells are subjected becomes unsustainable, there is a reduction in ΔΨ accompanied by a drastic reduction in intracellular ROS, as previously demonstrated in other cellular systems [62].

## 5. Conclusions

In conclusion, the variation in CPA concentrations and exposure times allowed the study to obtain new results that could support and guide further studies leading to the optimization of an immature COC vitrification protocol in pre-pubertal lambs. In the present study, the combination of low concentrations of DMSO and EG associated with prolonged exposure times gave slightly more encouraging results in balancing CPA penetration with minimal toxicity during vitrification procedures. Further studies are necessary to evaluate the development competence of immature COCs vitrified through both tested protocols and to improve the vitrification protocol by fine tuning CPA concentrations and exposure times as well as the addition of antioxidant molecules, hormones or growth factors in both vitrification and IVM media composition.

## Data Availability

The data presented in this study are available on request from the corresponding author.
